# Organizational Readiness to Implement Community Pharmacy-Based Opioid Counseling and Naloxone Services: A Scoping Review of Current Practice Models and Opportunities

**DOI:** 10.3390/pharmacy11030099

**Published:** 2023-06-11

**Authors:** Lindsey Hohmann, Klaudia Harris, Yi Zhao, Karen Marlowe, Haley Phillippe, Chris Correia, Brent Fox

**Affiliations:** 1 Department of Pharmacy Practice, Harrison College of Pharmacy, Auburn University, 1330 Walker Building, Auburn, AL 36849, USA; kkh0019@auburn.edu (K.H.); marlokf@auburn.edu (K.M.); phillh1@auburn.edu (H.P.); 2 Department of Health Outcomes Research and Policy, Harrison College of Pharmacy, Auburn University, 4306 Walker Building, Auburn, AL 36849, USA; yzz0167@auburn.edu (Y.Z.); foxbren@auburn.edu (B.F.); 3 Department of Psychological Sciences, College of Liberal Arts, Auburn University, 221 Cary Hall, Auburn, AL 36849, USA; correcj@auburn.edu

**Keywords:** opioid counseling, naloxone service, community pharmacy, implementation

## Abstract

The purpose of this study was to explore existing practice models and opportunities surrounding community pharmacist-delivered opioid counseling and naloxone (OCN) services in the U.S., with the goal of enhancing organizational readiness and improving patient access. A scoping literature review was conducted. English-language articles published in peer-reviewed journals from January 2012–July 2022 were sought via PubMed, CINAHL, IPA, and Google Scholar using permutations of terms such as “pharmacist/pharmacy”, “opioid/opiate”, “naloxone”, “counseling”, and “implement/implementation”. Original articles reporting the resources/inputs (personnel; pharmacist full-time equivalents; facilities and expenses; in-house versus outsourced personnel), implementation processes (legal source of pharmacist authority; patient identification strategies; intervention procedures; workflow strategies; business operations), and programmatic outcomes (uptake and delivery; interventions made; economic impact; patient or provider satisfaction) of pharmacist-delivered OCN services in community (retail) settings were retained. Twelve articles describing ten unique studies were included. The studies primarily used quasi-experimental designs and were published from 2017 to 2021. The articles described seven broad program elements/themes: interprofessional collaboration (*n* = 2); patient education format including one-on-one patient education (*n* = 12) and group education sessions (*n* = 1); non-pharmacist provider education (*n* = 2); pharmacy staff education (*n* = 8); opioid misuse screening tools (*n* = 7); naloxone recommendation/dispensing (*n* = 12); and opioid therapy and pain management (*n* = 1). Pharmacists screened/counseled 11–2716 patients and provided 11–430 doses of naloxone. Limited implementation costs, patient/provider satisfaction, or economic impact measures were reported. This review may serve as a guide for community pharmacists in implementing OCN services in their own practices. Future studies should clarify OCN program implementation costs, patient/provider satisfaction, and the economic impact.

## 1. Introduction

Opioid misuse is a major public health issue in the United States. Opioids are a class of medications that include drugs such as oxycodone, hydrocodone, codeine, and morphine [1]. Of the 92,000 drug overdose deaths recorded in 2020 in the US, 75% of those involved an opioid [2]. Furthermore, it is estimated that 44 people die every day from overdoses involving prescription opioids [3]. In recent years, the Centers for Disease Control and Prevention (CDC) noted that drug overdose deaths are rising in both rural and urban areas [4], posing a significant economic burden, with the U.S economic cost of opioid use disorder (OUD) and fatal opioid overdose during 2017 totaling USD 1021 billion [5]. Thus, it is increasingly important that healthcare providers are ready to combat this epidemic.

Community pharmacists are well placed to take the lead in combatting the opioid epidemic by providing access to naloxone, the opioid overdose antidote [6]. All U.S. states have enacted naloxone access laws, allowing pharmacists to provide naloxone without a physician visit via statewide standing orders, protocols, or pharmacist prescriptive authority, depending on the state [7,8]. Community pharmacy-based opioid counseling and naloxone services (OCN) may include education on opioid dosing and side effects, risks of overdose, signs and symptoms of overdose, overdose management, the identification of opioid compounds, and naloxone recommendations, dispensing, and counseling. Pharmacy-based OCN services have been shown to increase patient awareness of overdose risk, increase patient access to naloxone, and decrease opioid overdose mortality [9,10,11,12,13,14]. Despite this, naloxone dispensing is not at capacity, with only 1 naloxone prescription dispensed out of every 69 high-dose opioid prescriptions in 2018 [12]. With the U.S. Food and Drug Administration’s approval of the over-the-counter (OTC) status of the 4 mg naloxone nasal spray (Narcan^®^) on 29 March 2023 [15], methods for enhancing community pharmacy-based OCN services and bringing them up to capacity are even more critical.

However, little is known about pharmacists’ organizational readiness to implement OCN services. Previous research has focused on improving pharmacist knowledge and training related to naloxone [16], pain management [17], the treatment of opioid use disorder [18], and enhancing naloxone dispensing [16]. Limited research has explored the structures and processes underlying community pharmacy-based naloxone services implementation, including the service workflows currently being utilized, the resources used, and the cost of implementation [19]. Understanding these factors is the first step in identifying best practices and actionable gaps in practice that can be leveraged to enhance OCN services. Furthermore, given the recent approval of Narcan’s^®^ OTC status [15], leveraging the best practices and identifying actionable gaps is more urgent than ever in order to prepare pharmacists for potential increases in naloxone inquiries from their patients. Therefore, the purpose of this study was to explore the existing practice models and practice opportunities surrounding community pharmacist-delivered opioid counseling and naloxone services in the United States, with the goal of enhancing organizational readiness and improving patient access.

## 2. Review Process

In order to broadly explore current community pharmacy-based OCN practice models and opportunities for service enhancement, a scoping review of the literature was conducted. The review methodology, including data extraction and data synthesis, was informed by scoping review recommendations published by Arksey and O’Malley [20] and Levac and colleagues [21].

### 2.1. Data Sources and Search Terms

A broad search of the literature was conducted using the PubMed, CINAHL, IPA, and Google Scholar online databases in July 2022. A priori search terms were chosen in order to best characterize the development, implementation, and current operations of community pharmacy-based opioid counseling and naloxone (OCN) services. Community pharmacies were defined as retail pharmacies that serve the public. In order to encompass a wide range of service/program models, OCN services were loosely defined as any naloxone dispensing, naloxone recommendations, naloxone education, or illicit/prescription opioid counseling provided by pharmacy personnel to people who use opioids, their caregivers, or other healthcare providers. Searches included permutations of terms such as: “pharmacist/pharmacy”, “opioid/opiate”, “naloxone”, “counseling”, “program”, “resource”, “process”, “intervention”, “financial”, “satisfaction”, “develop/development”, and “implement/implementation” (Table 1) [22]. Searches were limited to English-language articles published between January 2012 (when pharmacy naloxone access laws began in the U.S.) and July 2022. Reference lists of published studies were also manually searched to find additional articles that may have been missed in database searches.

### 2.2. Study Selection, Outcome Measures, and Data Extraction

Studies were eligible for inclusion in the scoping review if they used an experimental, quasi-experimental, or observational study design, described a community pharmacy-based OCN program/service, and reported at least one outcome measure of interest with extractable data. Outcome measures and categories of interest included: (1) resources and inputs involved in community pharmacy-based OCN program development (personnel; pharmacist full-time equivalents; facilities and expenses; in-house versus outsourced personnel); (2) program/service implementation processes (legal source of pharmacist authority; patient identification strategies; intervention procedures; workflow strategies; business operations); and (3) programmatic outcomes (uptake and delivery; interventions made; economic impact; patient or provider satisfaction). Outcome measures were broadly guided by the Donabedian Model for Quality of Care, which postulates that an organization’s structures (resources and inputs) influence its processes (implementation processes), which in turn influence outcomes (programmatic outcomes) [23,24]. Categories within resources/inputs (e.g., personnel), implementation processes (e.g., patient identification strategies), and programmatic outcomes (e.g., economic impact) measures were further informed by previous work by Hohmann et al. [22] and the ECHO (economic, clinical, and humanistic outcomes) Model [25]. Studies describing programs that were not pharmacist-delivered (e.g., delivered by a nurse or other healthcare professional), conducted outside of the United States, or conducted in a setting other than a community pharmacy were excluded from the review.

Searches were conducted by two investigators (L.H., K.H.), beginning with the title and abstract review, followed by a full text review and hand-searching of reference lists. The final articles included in the review were agreed upon by the research team (B.F., H.P., K.M., C.C., Y.Z.), which included a diverse array of content experts, as recommended by Arksey, O’Malley, Levac, and colleagues [20,21], with discrepancies resolved via discourse and consensus. Data were extracted by two investigators (L.H., K.H.) using a standardized template, including the study design, setting, study period, study population, and outcomes variables. EndNote version X9 (Clarivate™, Philadelphia, PA, USA) citation management software was used as a data organization tool and to assist with the removal of duplicate articles.

### 2.3. Data Synthesis

The final retained articles were assessed using a qualitative narrative synthesis technique to identify broad OCN program elements/themes and summarize outcome measures of interest. Specifically, following a precedent set by Nielsen and Van Hout [26], a qualitative content analysis process was used to inductively identify core OCN program elements across studies. Subsequent to this initial round of content analysis, core program elements were revised to create final program themes and sub-themes. Additionally, resources/inputs, implementation processes, and programmatic outcomes data were deductively grouped into each of the aforementioned pre-determined categories informed by Hohmann et al. [22] and the ECHO model [25] (e.g., workflow strategies; see Section 2.2) and then inductively organized into meaningful sub-categories in order to provide a broad picture of the “building blocks” involved in OCN service development and implementation. Data synthesis was performed by a single investigator (L.H.) in consultation with the research team, and the final results were agreed upon by all members. 

## 3. Results

**Figure 1:**
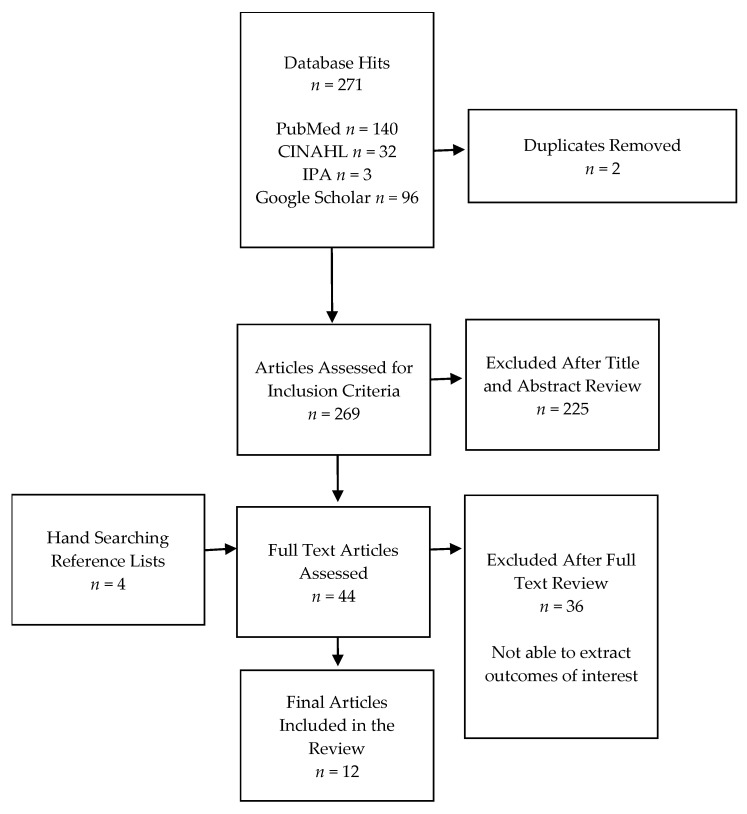
Flow diagram of study selection.

A total of 271 database hits were obtained, and 269 articles were assessed for inclusion criteria via the title and abstract review after the removal of duplicates (Figure 1). Forty-four full-text articles were screened for eligibility, with an additional four articles obtained by hand-searching reference lists. Twelve articles describing ten unique studies representing current community pharmacist-delivered opioid counseling and naloxone (OCN) practices were retained in the final review [9,10,14,19,27,28,29,30,31,32,33,34].

### 3.1. Study Characteristics

Studies meeting the inclusion criteria for the review were published between 2017 and 2021 and primarily used quasi-experimental (*n* = 8) and observational (*n* = 3) study designs, with a single experimental design (Table 2). One-group (single-arm) studies were predominant (*n* = 9), including pretest–posttest (*n* = 5) [9,29,30,31,33], posttest (*n* = 1) [34], and retrospective cohort (*n* = 3) [10,19,28] designs. Two-group study designs included non-randomized controlled trials (*n* = 2) [14,32] and a single randomized controlled trial (*n* = 1) [27]. The largest number of articles (*n* = 4) described studies conducted in North Dakota, with others taking place in Pennsylvania (*n* = 2), Arkansas (*n* = 1), California (*n* = 1), North Carolina (*n* = 1), Ohio (*n* = 1), Washington (*n* = 1), and West Virginia (*n* = 1). The patient population served by each community pharmacy-based OCN program included all patients receiving opioid therapy (*n* = 6), patients receiving opioid therapy and at a high risk of overdose (*n* = 4), potential bystanders to an overdose situation (*n* = 1), and those receiving buprenorphine-containing products for the treatment of opioid use disorder (OUD) (*n* = 1).

### 3.2. Program Themes

The articles described seven broad program elements/themes (Table 3): (1) interprofessional collaboration (*n* = 2); (2) patient education format including two sub-themes of one-on-one patient education (*n* = 12) and group education sessions (*n* = 1); (3) non-pharmacist provider education (*n* = 2); (4) pharmacy staff education (*n* = 8); (5) opioid misuse screening tools (*n* = 7); (6) naloxone recommendation/dispensing (*n* = 12); and (7) opioid therapy and pain management (*n* = 1).

#### 3.2.1. Interprofessional Collaboration

Two articles described community pharmacy-based OCN services incorporating elements of interprofessional collaboration. Specifically, Akers and colleagues [10] partnered with the Seattle-King County Public Health Department to create a collaborative drug therapy agreement (CDTA) for naloxone and to identify other organizations providing naloxone in their local area. They also provided ready-made naloxone prescription templates and order sets to local prescribers to engage them in the service. Furthermore, Manzur and colleagues [28] described a collaborative OCN service between a community pharmacy and a nearby rheumatology clinic within an academic medical center, whereby clinic patients prescribed opioids for chronic pain and at a perceived high risk of overdose according to Centers for Disease Control and Prevention (CDC) guidelines [35] were referred to the pharmacy for opioid medication counseling, naloxone counseling, and the provision of naloxone. Pharmacist recommendations regarding therapy were then relayed to the prescriber prior to the patient’s next clinic appointment.

#### 3.2.2. Patient Education Format: One-on-One Patient Education versus Group Education Sessions

The patient education format was divided into two sub-themes describing distinct service models: one-on-one education sessions and group education sessions. All articles [9,10,14,19,27,28,29,30,31,32,33,34] described a one-on-one (individual) patient education component of their OCN service, while only one article (Akers et al.) [10] discussed the provision of group education sessions. Notably, individual education sessions primarily focused on counseling regarding naloxone administration and how to recognize and manage an opioid overdose. Materials such as checklists, pamphlets, and posters used in these individual counseling sessions were adapted from the Substance Abuse and Mental Health Services Administration (SAMHSA) toolkit [36] and templates available from Prescribe to Prevent [37] and the Maximizing OpiOid Safety with Naloxone (MOON) study [38]. Some studies mentioned the incorporation of video training demonstrating naloxone administration techniques into individual sessions; for example, Akers and colleagues cited publicly available You Tube videos [10]. In terms of group education sessions, Akers and colleagues [10] described group sessions provided to the community that focused on several elements surrounding opioid overdoses, including: (1) how to manage an overdose situation; (2) how to train others; (3) statistics; and (4) dispelling myths.

#### 3.2.3. Non-Pharmacist Provider Education

Two studies reported multidisciplinary healthcare provider education (education of providers outside of the pharmacy) as part of their OCN service [10,28]. For example, Akers and colleagues [10] discussed the implementation of opioid overdose and naloxone education for local physicians with practice sites near the community pharmacy in Washington State. In addition, providers were given ready-to-use naloxone prescription templates from Prescribe to Prevent [37] and directed to the Interagency Guideline of Prescribing Opioids for pain from the Washington State Agency Medical Directors Group [39], which outlined how to identify patients with risk factors for opioid overdoses. Similarly, Manzur and colleagues [28] mentioned providing education to prescribers located at a nearby rheumatology clinic, including information regarding naloxone administration, pain management strategies (pharmacologic, non-pharmacologic, lifestyle), interactions, and side effects.

#### 3.2.4. Pharmacy Staff Education

Eight articles described training pharmacy personnel regarding opioid overdose management and naloxone as part of their OCN service [10,19,29,30,31,32,33,34]. Of these, two articles discussed using national, publicly available sources to adapt and inform their training materials. Specifically, Wilkerson and colleagues [19] utilized training videos from Prescribe to Prevent [39], and Akers and colleagues [10] used the SAMHSA toolkit [36] to inform their training checklist. Five articles utilized statewide experts or sources to develop their training, while two used local and intraorganizational sources. For example, in terms of statewide experts and sources, Skoy and colleagues [29,30] and Strand and colleagues [31,34] developed their pharmacist training program in collaboration with faculty from North Dakota State University and state public health officials, and Santa and colleagues [33] utilized an SBIRT (screening, brief intervention, and referral to treatment) training previously developed by the University of Pittsburgh. At the local and intraorganizational level, Sexton and colleagues [32] utilized training delivered by the pharmacy’s clinical pharmacist and resident, and Wilkerson and colleagues [19] used a training video developed by the pharmacy’s corporate team in addition to the aforementioned nationally sourced training. Furthermore, the largest number of articles described the provision of a hybrid (online and in-person) training format (*n* = 5) [29,30,31,33,34], followed by online only (*n* = 2) [10,19] and in-person only (*n* = 1) [32]. Three articles mentioned using video media to demonstrate opioid overdose management and naloxone administration [10,19,33], and one mentioned the use of a simulation [33]. Only Skoy and colleagues [29,30] and Strand and colleagues [31,34] reported the development of training specific for pharmacy technicians. The length of pharmacist training was infrequently reported, but in those articles in which it was discussed, training ranged from 3 h [34] to a full day [33], with continuing education credit offered.

#### 3.2.5. Opioid Misuse Screening Tools

Seven articles describe the usage of an opioid misuse screening tool when implementing community pharmacy-based OCN services [27,28,29,30,31,33,34]. Cochran and colleagues [27] screened patients for opioid misuse at the time of service using the Prescription Opioid Misuse Index (POMI) [40], which is explained as a brief six-item questionnaire that asks patients about behaviors related to their current use of opioid pain medication. The POMI is scored from 0 to 6, with scores of 2 or above indicating the potential for opioid misuse [40]. In contrast to a point-of-service screening, Skoy and colleagues [29,30] and Strand and colleagues [31,34] used an electronic or hardcopy opioid misuse screener (the Opioid Risk Tool (ORT) [41]) embedded in their patient intake form. Using the ORT, the risk of opioid misuse is scored from 0 to 26, with scores over 8 indicating a high risk and a greater need for naloxone [31]. This screening tool was provided to pharmacies in an Opioid Misuse Risk Prevention toolkit as part of the larger ONE Rx pharmacist opioid and naloxone education program in North Dakota [42]. Manzur and colleagues [28] also used the ORT as part of a comprehensive pain management assessment. Additionally, Santa and colleagues [33] utilized the SBIRT framework, which provides a guide for action including screening, brief intervention (patient counseling), and referral to treatment [43]; however, although it was mentioned that a formal opioid misuse screening tool was used, no further information regarding the screening tool was provided.

#### 3.2.6. Naloxone Recommendation/Dispensing

All studies incorporated naloxone recommendations into their pharmacy-based OCN services [9,10,11,19,27,28,29,30,31,32,33,34]. The largest number of articles (*n* = 5) [9,10,19,32,34] reported using educational brochures, pamphlets, or handouts to guide their naloxone recommendations to patients, while very few mentioned using video presentations (*n* = 1) [11] or naloxone demonstration kits (*n* = 1) [9]. Neither Cochran and colleagues [27] nor Manzur and colleagues [28] reported the use of any patient education materials in guiding their naloxone counseling. Although Santa and colleagues [33] likewise did not mention the use of patient-facing naloxone education materials, they utilized a workflow outline to evaluate and monitor the pharmacy’s naloxone dispensing on a weekly basis. Few studies discussed the organization/storage of naloxone educational materials in the pharmacy; those that did (*n* = 3) [29,30,31] mentioned the collation of all printed materials in a binder stored in the pharmacy as well as a dedicated website (https://one-program.org/, accessed on 8 February 2023) [44] for organizing and archiving naloxone educational materials for future printing. 

#### 3.2.7. Opioid Therapy and Pain Management

Only one study reported conducting comprehensive opioid therapy and pain management in a community pharmacy setting [28]. Specifically, Manzur and colleagues [28] assessed patients’ pain management using a numeric pain rating scale and the Pain, Enjoyment, General Activity (PEG) tool [44] as well as associated concomitant disease states including mental health using the Patient Health Questionnaire-9 (PHQ-9) [45]. Pharmacists provided medication recommendations including opioid dose adjustments, the addition of adjuvant therapy, and laboratory tests to the provider who referred the patient to their service. As part of the assessment, they performed opioid risk mitigation strategies including screening for potential misuse using the ORT, PDMP review, opioid and non-opioid pain medication counseling, pain management education (pharmacologic, non-pharmacologic, lifestyle), naloxone education, and naloxone co-prescribing.

### 3.3. Program Inputs and Resources

All studies utilized in-house (versus outsourced) pharmacists to perform OCN services; the utilization of pharmacy technicians (*n* = 3) [10,19,32], student pharmacists (*n* = 2) [19,32], and pharmacy residents (*n* = 2) [9,28] was also reported in a limited number of studies (Table 4). Few studies (*n* = 2) reported pharmacist full-time equivalents (FTEs), but among those that did, the mean pharmacist FTEs dedicated to OCN services was 1.5 [10,27]. Additionally, a few articles (*n* = 3) described the pharmacy facilities dedicated to OCN services. Specifically, Manzur and colleagues [28] and Hines and colleagues [9] reported using a private counseling/exam room to conduct OCN services. Akers and colleagues described a process of “rooming” patients prior to counseling, but no further information regarding the facilities was provided [10]. None of the included articles discussed the expenses incurred in OCN implementation.

### 3.4. Program Implementation Processes

The implementation processes utilized in community pharmacy-based OCN were divided into five broad categories (Table 5): (1) pharmacist authority; (2) patient identification; (3) pharmacist interventions; (4) workflow; and (5) business operations.

#### 3.4.1. Pharmacist Authority

Eleven articles reported the type of pharmacist authority utilized to provide OCN. The largest number of articles reported the use of a statewide naloxone standing order (*n* = 4) [9,14,32,33] or pharmacist prescriptive authority (*n* = 4) [29,30,31,34]. Protocols with local physicians (*n* = 1) [19] and collaborative drug therapy agreements (CDTA) (*n* = 1) [10] were also mentioned. Manzur and colleagues specifically mentioned that they did not use a collaborative practice agreement because their OCN service was strictly consultative [28].

#### 3.4.2. Patient Identification

In terms of identifying patient recipients of OCN services, a variety of approaches and communication strategies were described, including general/passive advertisements (posters or flyers displayed in the pharmacy), targeted offers (offering naloxone only to those at increased risk of an opioid overdose based on certain criteria) with or without the use of a screening tool, and universal offers (offering naloxone to all patients prescribed opioids). The majority of articles described using a targeted approach (*n* = 7) [27,28,29,30,31,32,34], with few using universal (*n* = 2) [9,33] or general/passive approaches (*n* = 1) [10]. Within studies using a targeted approach, some utilized criteria from the CDC Opioid Prescribing Guidelines [35] to determine the risk for an opioid overdose [14,28,32], while others used guidelines set forth by specific screening tools (see the Opioid Misuse Screening Tools sub-section above). Two studies reported using a combination of targeted and general/passive approaches [14,19]. Furthermore, two studies discussed the use of technology in patient identification. Specifically, Sexton and colleagues [32] and Teeter and colleagues [14] reported the use of “clinical flags” in the pharmacy dispensing software to alert the pharmacist to patients at an increased risk of an opioid overdose or harm from opioids based on pre-determined criteria such as morphine milligram equivalents (MME) and concomitant medications.

#### 3.4.3. Pharmacist Interventions

The types of services/interventions offered in community pharmacies primarily consisted of opioid education and naloxone dispensing (OEND) (*n* = 9) [9,14,19,29,30,31,32,33,34], with specialized services offered at a few pharmacies, including take-home naloxone (THN) plus an extensive multidisciplinary education program (*n* = 1) [10], pain medication management (*n* = 1) [28], and a brief motivational intervention plus medication therapy management (BMI-MTM) (*n* = 1) [27]. Two studies reported the use of motivational interviewing (MI) concepts in their service/intervention. Specifically, the BMI-MTM intervention performed by Cochran and colleagues incorporated concepts of MI in a pharmacist–patient consultation [27], while Santa and colleagues utilized MI during pharmacist–patient interactions as part of the SBIRT framework guiding their program [33]. Furthermore, Skoy and colleagues [29,30] and Strand and colleagues [31,34] offered additional services as part of their OEND programs, including drug take-back. Although the aforementioned authors [29,30,31,34], as well as Santa and colleagues [33], also mentioned referral of patients to community resources for further treatment, specific details regarding the referral process were not reported beyond the provision of a list of local providers.

#### 3.4.4. Workflow

Community pharmacy-based OCN services/interventions ranged from 5 to 45 min in length [28,29], with most offered in-person (*n* = 9) [9,10,14,19,28,29,32,33,34] and on a walk-in basis (*n* = 6) [9,14,19,29,32,34]. Few pharmacies utilized an appointment-based (*n* = 2) [27,28] service model, and none used a solely telephonic model. Two articles reported using hybrid service models. Specifically, Cochran and colleagues provided both in-person and telephone-based services [27], while Akers and colleagues provided services on both a walk-in and appointment basis [10]. Additionally, nine articles reported using some sort of materials to assist with implementation of their OCN services, including patient intake forms [10], workflow checklists [32,33], instructions posted at each pharmacy workstation [32], and decision-making tools or guides [14,29,30,31,34]. In particular, Skoy and colleagues [29] and Strand and colleagues [31] reported using a unique implementation toolkit that included a patient screening form accessed via a mobile device using a QR code, a tablet available at the pharmacy, or paper.

#### 3.4.5. Business Operations

Five of the twelve articles reported the utilization of marketing materials or campaigns to increase patient demand for OCN services. Specifically, Skoy and colleagues [29] and Strand and colleagues [31,34] used a comprehensive marketing campaign including television and newspaper interviews, emails from their state board of pharmacy, window clings, posters, brochures, and pharmacist buttons. Wilkerson and colleagues [19] used signs advertising the availability of naloxone posted outside the pharmacy, while Teeter and colleagues [14] utilized posters derived from the MOON study website [38] that were posted inside the pharmacy in the waiting room and at the pick-up counter and rotated weekly. Furthermore, while seven articles reported the use of formalized policies and procedures to guide their OCN services (e.g., clarification of roles, workstation duties, etc.) [10,19,29,31,32,33,34], only one article described using a formalized process to evaluate OCN service fidelity (how closely the service followed the formal protocol) [33]. In addition, few articles (*n* = 3) [9,10,14] reported details regarding their service reimbursement model (disregarding cases where reimbursement came from grant funding alone). Specifically, Teeter and colleagues [14] described naloxone product reimbursement via third-party insurance billing for brand Narcan^®^ or generic naloxone vials with a nasal atomizer. Similarly, Hines and colleagues [9] utilized third-party insurance billing, primarily Medicaid, to reimburse naloxone product costs, although they only stocked and dispensed the Narcan^®^ nasal spray due to the greater likelihood of insurance coverage for this formulation. Akers and colleagues [10] utilized an out-of-pocket reimbursement model incorporating the costs of two naloxone doses, a counseling fee, a nasal atomizer, and a breathing mask.

### 3.5. Programmatic Outcomes

Pharmacists screened/counseled between 11 [28] and 2716 [30] patients and provided 11 [32] to 430 [33] doses of naloxone (Table 6). No studies reported measures of program economic impact (e.g., revenue generated, return-on-investment), and few reported patient/provider satisfaction measures (*n* = 2) [14,27]. Specifically, Cochran and colleagues [27] measured patient satisfaction via post-program surveys using a Likert-type scale and found that 92.4% of participants were satisfied with the program, with a mean program rating of 4.2 out of 5. Furthermore, Teeter and colleagues [14] performed post-program interviews with pharmacy personnel to assess program feasibility, acceptability, and appropriateness; all measures were discussed positively. Two articles reported anecdotal evidence of program satisfaction but did not report a priori measures of satisfaction via surveys, interviews, or other methods [10,32].

## 4. Discussion

This scoping review used evidence-based reporting guidelines [20,21] to explore the existing practice models and practice opportunities surrounding community pharmacist-delivered opioid counseling and naloxone services in the United States, with the goal of enhancing organizational readiness and improving patient access. This review fills a gap in the harm reduction literature, as limited reviews have examined opioid counseling and naloxone services in the community pharmacy setting [26,46], and to the authors’ knowledge, this is the first review exploring community pharmacy-based OCN implementation inputs and processes in addition to outputs and outcomes. Overall, a wide variety of community pharmacy-based OCN program themes were identified throughout the articles included in this review, with several opportunities for growth identified that are related to program inputs and resources, implementation processes, and programmatic outcomes.

Specifically, seven broad program themes were identified. The themes and sub-themes occurring in the largest number of articles (*n* = 12) included one-on-one patient education and naloxone recommendation/dispensing. On the other hand, opioid therapy and pain management and group education sessions were cited the least frequently (*n* = 1). Based on this, it is evident that one-on-one patient education and naloxone recommendation/dispensing are common, widely accepted elements of community pharmacy-based OCN practice models. With that being said, there is an opportunity for community pharmacies to implement group opioid/naloxone education sessions and opioid therapy management services based on the precedents set by Akers et al. [10] and Manzur and colleagues [28]. In particular, given that group education sessions are equally efficacious yet more cost-effective compared to one-on-one sessions [47,48], this represents an effective but underutilized service that can be adopted by independently owned community pharmacies to distinguish their business in the current competitive market. Pharmacists wishing to incorporate these elements may start by contacting their local health departments and local physician offices to initiate partnerships and/or referral networks [49].

The current review found that, in general, community pharmacy-based OCN program inputs and resources were not well described. The most commonly discussed program input involved the type of personnel/staffing used to perform the service, with in-house pharmacists being the norm across all articles. However, given that few articles described incorporating non-pharmacist personnel into their OCN service [9,10,19,28,32], there is an opportunity for community pharmacies to further utilize pharmacy technicians, interns, and residents. In light of the increasing roles of pharmacy technicians in other services such as immunizations [50] and medication therapy management (MTM) [51], this represents a potential to maximize OCN service return-on-investment while freeing up pharmacists’ time by assigning non-pharmacist staff to perform non-counseling tasks such as patient intake, screening, naloxone dispensing (as applicable), rooming the patient, and post-visit paperwork. Furthermore, no articles described the use of an outsourced OCN service provider, such as a clinical pharmacist who floats between multiple stores, which represents an area for further exploration and may open up novel job opportunities in the pharmacy profession, potentially increasing pharmacist job satisfaction [52]. Along the same lines, few studies reported pharmacist FTEs involved in OCN service implementation [10,27] or pharmacy facilities necessary to perform the service [9,28], and none discussed expenses incurred in initiating and implementing OCN, making it difficult to come to any firm conclusions regarding the minimum number of pharmacists, types of facilities (consultation room, pick-up window, etc.), or financial investments that are required for a successful service. Following the FDA’s approval of the OTC sale of Narcan^®^, and taking into account that other formulations of naloxone will remain prescription-only, understanding the facilities and expenses incurred in stocking and furnishing naloxone is more important than ever [15]. However, it is important to note that, before issues of outsourcing personnel and start-up costs can be addressed, larger ongoing barriers to community pharmacy-based OCN implementation must be acknowledged and overcome. Specifically, pharmacists consistently report a lack of time, staff shortages, competing workflow priorities, and a lack of knowledge/training about opioids and naloxone as barriers to OCN service implementation [16,53,54]. Gaining management support to provide additional support staff and protected time for training during work hours through the use of an OCN service “champion” may assist community pharmacy personnel in taking the first step towards mitigating these barriers [55,56]. Future studies should consider exploring these issues.

Compared to program inputs and resources, program implementation processes were more fully described by the included articles. For example, current successful community pharmacy-based OCN services appear to utilize in-person walk-in models [9,14,19,29,32,34] making use of statewide naloxone standing orders or prescriptive authority [9,14,29,30,31,32,33,34], with targeted patient identification strategies (e.g., based on CDC guidelines or other screening tools) [27,28,29,30,31,32,34] and workflow aids such as checklists, decision guides, and instruction sheets at each workstation. However, gaps and opportunities still exist. Specifically, few pharmacies utilized an appointment-based model for OCN services [27,28]. Given that appointment-based models have been shown to improve patient health outcomes (including medication adherence), increase the number of prescription fills, and reduce the number of unnecessary trips to the pharmacy [57], the use of this model may help some pharmacies to overcome commonly reported barriers to OCN services implementation, including inconsistent reimbursement and a lack of time for performing services [16,58]. Furthermore, pharmacies encountering patient resistance to OCN services may consider using a universal rather than a targeted approach to patient identification. Although few studies in the current review reported using a universal approach to patient identification and communication, this approach has been shown to reduce perceptions of stigma and targeting experienced by patients [59,60]. The use of technology in patient intake/screening (e.g., tablets, QR codes) [29,31] and identification (e.g., clinical flags in the dispensing software) [14,32] has likewise been reported in few articles and represents an area for future research in the OCN services realm, as the integration of health information technology has proven successful in other services such as MTM [61]. Lastly, although current OCN implementation processes were generally well described in most areas, business operations were not as clear. In particular, less than half of the included articles described their OCN program marketing strategies [14,19,29,31,34], and few described their service reimbursement model [9,10,14] or processes for evaluating service fidelity [33]. Of those that described their service reimbursement model, both an out-of-pocket model including a counseling fee [10] as well as third-party models focused solely on naloxone product reimbursement were reported [9,14]. Pharmacies wishing to initiate or enhance their OCN services implementation may benefit from conducting a needs assessment of their client population to determine which reimbursement model is the most feasible and acceptable [62], particularly when OTC sales of Narcan^®^ are initiated [15]. Additionally, pharmacy personnel planning to implement OCN services may consult Supplementary Table S1 for a collated list of OCN resources and tools mentioned throughout this review.

In terms of programmatic outcomes, the majority of included studies reported positive effects of community pharmacy-based OCN services. Specifically, OCN services resulted in the education of thousands of patients, the provision of hundreds of doses of naloxone, opioid/pain medication-related problem identification, and associated potential decreases in opioid overdose deaths. Furthermore, few studies measured patient/provider satisfaction with OCN services, but those that did observed positive results. Given that quantified measures of patient experiences can help to identify service limitations [63], satisfaction measures should be further examined in future studies in order to improve intervention quality and service delivery. Lastly, no studies in this review evaluated economic outcomes resulting from community pharmacy-based OCN services, and in fact, limited information regarding the economic impact exists [64]. Acharya and colleagues demonstrated the positive cost-effectiveness of a pharmacist-based intranasal naloxone distribution intervention using a modeling process known as a Markov model [65]. However, evaluations of the economic effects (e.g., return on investment, healthcare expenditures averted, etc.) of community pharmacy-based OCN are needed in future research in order to advance the profession of pharmacy and demonstrate the value of pharmacy services.

### Limitations

Several limitations of this review must be taken into account. First, although the current study identified literature from a variety of online databases including PubMed, CINAHL, IPA, and Google Scholar, we may have missed additional relevant studies present in other databases such as ClinicalTrials.gov. Second, this study only included peer-reviewed articles. Hence, on-going work that was not yet published, available in pre-print servers, or published in non-peer-reviewed editorials or websites at the time the search was conducted is not included in this review. This introduces an element of publication bias; however, the use of the aforementioned databases and peer-reviewed articles was sufficient to achieve the purpose of this scoping review: gaining a broad understanding of the community pharmacy-based OCN landscape and identifying gaps and opportunities. Third, this study was not able to report international differences in OCN programs since studies published outside of the United States and in languages other than English were excluded. Lastly, given the nature of this study as a scoping review, the quality of articles was not assessed; future systematic reviews focused on a similar topic may wish to assess article quality, although the wide variety of measures and outcomes utilized in each article may complicate this assessment.

## 5. Conclusions

This review may serve as a guide for community pharmacists in implementing OCN services in their own practices, highlighting areas for organizational enhancement. There is an opportunity for community pharmacists to utilize ancillary pharmacy staff, including technicians, students, and residents, to improve OCN workflow efficiency. Interprofessional collaborations and group education sessions are little-utilized program elements that could increase uptake. Future studies should clarify OCN program implementation costs, patient/provider satisfaction, and economic impact.

## Figures and Tables

**Table 1:** Example search string.

Database ^a^	Key Words
PubMed	(pharmacist or pharmacy) and ((opioid or opiate) or naloxone) and (counseling or service or program) and (resource or input or personnel or process or workflow or intervention or financial or economic or satisfaction) and ((develop or development) or (uptake or delivery) or planning) and (implement or implementation)

^a^ Limits: English, 2012–2022.

**Table 2:** Study characteristics.

No.	Study	Study Design	Setting	Study Period	Study Population
1	Akers et al., 2017 [10]	Program evaluation, single-arm retrospective cohort	Community pharmacy. Kelley-Ross Pharmacy Group, Seattle, WA.	2012–2016	Bystanders (family and friends), median age 57 years
2	Cochran et al., 2019 [27]	Randomized controlled trial (RCT)	Two community pharmacies located in southwestern Pennsylvania, one associated with an academic medical center and the other an independent pharmacy in a rural county.	2017–2018	Adults aged 18 or older receiving prescription opioid therapy.
3	Manzur et al., 2020 [28]	Program evaluation, single-arm retrospective cohort	Community pharmacy in an academic medical center, CA.	2016–2018	Patients enrolled were prescribed opioids for chronic pain by a rheumatology clinic and at a high risk of an opioid overdose
4a	Skoy et al., 2020a [29]	Program evaluation, one-group pretest–posttest (pre–post intervention)	Community pharmacy in North Dakota.	2018–2019	All patients prescribed opioids
4b	Skoy et al., 2020b [30]	Program evaluation, one-group pretest–posttest (pre–post intervention)	Community pharmacy in North Dakota.	2018–2019	All patients prescribed opioids
4c	Strand et al., 2020 [31]	Program evaluation, one-group pretest–posttest (pre–post intervention)	A total of 149 community pharmacies in North Dakota.	2018–2019	All patients prescribed opioids
5	Strand et al., 2019 [34]	Program evaluation, one-group posttest (post-intervention)	A total of 11 independent community pharmacies in North Dakota.	2017–2018	All patients prescribed opioids
6	Wilkerson et al., 2020 [19]	Program evaluation, single-arm retrospective cohort	Kroger community pharmacies, Ohio. A total of 114 pharmacies in the Columbus Division and 102 pharmacies in the Cincinnati Division.	2016–2018	Individuals prescribed opioids and at a high risk of an opioid overdose, or those who request naloxone.
7	Hines et al., 2020 [9]	Program evaluation, one-group pretest–posttest (pre–post intervention)	Independent community pharmacy in West Virginia.	2 January 2019 to 15 February 2019	Patients receiving buprenorphine-containing prescriptions for opioid use disorder (OUD).
8	Sexton et al., 2019 [32]	Two-group non-randomized controlled trial	Two Kroger community pharmacies, North Carolina.	2017–2018	Individuals prescribed opioids and at a high risk of an opioid overdose.
9	Teeter et al., 2021 [14]	Two-group non-randomized controlled trial (explanatory sequential mixed-methods)	Two intervention pharmacies and two rural pharmacies within the Harps community pharmacy chain, Arkansas	2019–2020	Individuals prescribed opioids and at a high risk of an opioid overdose.
10	Santa et al., 2021 [33]	One-group pretest–posttest (pre–post educational intervention)	A total of 11 community pharmacies (chain and independent) in Philadelphia	July 2019–December 2019	All patients prescribed opioids

**Table 3:** Program themes.

Study	Interprofessional Collaboration	Patient Education Format		Non-Pharmacist Provider Education	Pharmacy Staff Education	Opioid Misuse Screening Tool	Naloxone Recommendation/Dispensing	Opioid Therapy and Pain Management
		One-on-One Patient Education	Group Education Sessions					
Akers et al., 2017 [10]	x	x	x	x	x		x	
Cochran et al., 2019 [27]		x				x	x	
Manzur et al., 2020 [28]	x	x		x		x	x	x
Skoy et al., 2020a [29]		x			x	x	x	
Skoy et al., 2020b [30]		x			x	x	x	
Strand et al., 2020 [31]		x			x	x	x	
Strand et al., 2019 [34]		x			x	x	x	
Wilkerson et al., 2020 [19]		x			x		x	
Hines et al., 2020 [9]		x					x	
Sexton et al., 2019 [32]		x			x		x	
Teeter et al., 2021 [14]		x					x	
Santa et al., 2021 [33]		x			x	x	x	

**Table 4:** Program inputs and resources.

Study	Inputs and Resources		
	OCN Personnel	Pharmacist FTEs for OCN	OCN Facilities and Expenses
Akers et al., 2017 [10]	Pharmacist plus technicians and assistants	One	Patients were “roomed”; additional information not reported
Cochran et al., 2019 [27]	Staff pharmacist, study pharmacist, and navigator (researcher)	Two	Not reported
Manzur et al., 2020 [28]	Clinical pharmacist, pharmacy resident	Not reported	Patients seen in a private exam room in adjacent clinical suites of a community pharmacy
Skoy et al., 2020a [29]	Pharmacist	Not reported	Not reported
Skoy et al., 2020b [30]	Pharmacist	Not reported	Not reported
Strand et al., 2020 [31]	Pharmacist	Not reported	Not reported
Strand et al., 2019 [34]	Pharmacist	Not reported	Not reported
Wilkerson et al., 2020 [19]	Pharmacists, interns, technicians	Not reported	Note reported
Hines et al., 2020 [9]	Pharmacy resident	Not reported	Private counseling area
Sexton et al., 2019 [32]	Pharmacist, student pharmacist, technician	Not reported	Not reported
Teeter et al., 2021 [14]	Pharmacist	Not reported	Not reported
Santa et al., 2021 [33]	Pharmacist	Not reported	Not reported

**Table 5:** Program implementation processes.

Study	Pharmacist Authority	Patient Identification	Pharmacist Interventions	Workflow	Business Operations
Akers et al., 2017 [10]	Collaborative drug therapy agreement (CDTA) allowed pharmacists to initiate or modify therapy.	Communication strategy: General advertisement. Identification process: Technicians or clerks provide general information on program availability. Screening tool: not reported.	Type of service/intervention: Take-home naloxone program (THN) + education (individual, group, community, providers). Intervention details: Dispensed naloxone; provided education on naloxone administration and how to manage opioid overdoses.	Time: 20 min sessions. Setting: In-person. Materials: Patient intake form; video; standardized checklist. Model: Walk-in or appointment. Roles and processes: (Technician or clerk) Patient intake form completed. Patient roomed. (Pharmacist) Review pricing. Confirm preferred delivery method of naloxone for patient (IN or IM). (Technician or clerk) During video, prescription is processed by staff. (Pharmacist) Checklist reviewed with patient. (Technician or clerk) Naloxone dispensed and payment collected.	Marketing: Marketing to bystanders (details not reported). Formalized policies and procedures: Figure developed to clarify staff roles. External collaborators: Partnered with Seattle-King County Public Health Department for CDTA and naloxone distribution. Pricing/reimbursement model: Out-of-pocket pricing. Prices in internal documents for staff awareness. Price included two doses of naloxone, a counseling fee, and other materials (nasal atomizer, breathing mask, etc.).
Cochran et al., 2019 [27]	Not reported.	Communication strategy: Targeted offer. Identification process: Potential participants were approached in person by the pharmacist, technician, or researcher at the point-of-service if they were prescribed an opioid medication. Screening tool: Screened for prescription opioid misuse using the Prescription Opioid Misuse Index (POMI).	Type of service/intervention: Brief Motivational Intervention–Medication Therapy Management (BMI-MTM). Intervention details: Participants assigned to standard medication counseling (SMC) or SMC + BMI-MTM. BMI-MTM consisted of one pharmacist-led medication counseling/brief motivational session and eight weekly patient navigation sessions.	Time: 30–45 min. Setting: In-person (*n* = 1) and telephone (*n* = 8). Materials: Not reported. Model: Appointment. Roles and processes: (Pharmacist, technician, researcher) Screening. (Pharmacist) 30–45 min in-person BMI session. (Patient navigator who is a master’s level researcher) Eight patient navigation sessions via telephone; includes naloxone recommendation. (Pharmacist) Written summary of recommendations to patient and warm handoff to study navigator.	Not reported.
Manzur et al., 2020 [28]	No collaborative practice agreements. Consultation service with referring providers only.	Communication strategy: Targeted offer. Identification process: Rheumatology clinic patients prescribed opioids were identified by a community pharmacist or clinic provider as “high risk” and referred to the pilot program if they were prescribed (based on CDC guidelines): more than 1 short-acting opioid; more than 90 morphine milligram equivalents/day; more than 7 days’ supply of medications for acute pain; and high-risk medication combinations. Screening tool: Opioid Risk Tool (ORT).	Type of service/intervention: Pain medication management. Intervention details: Pharmacists assessed pain management and associated concomitant disease states and provided medication recommendations to referring provider. They also performed opioid risk mitigation strategies including ORT, naloxone education, naloxone prescription, PDMP review, pain score assessment, pain medication counseling, and pain management education.	Time: 45 min. Setting: In-person Materials: Not reported. Model: Appointment. Roles and processes: (Pharmacist or resident) Opioid risk mitigation strategies performed prior to prescriber visit.	Marketing: Not reported. Formalized policies and procedures: Not reported. External collaborators: Nearby rheumatology clinic. Pricing/reimbursement model: Grant-funded program; visits free for patients.
Skoy et al., 2020a ^a^ [29]	Pharmacist authority to prescribe naloxone in North Dakota since 1 April 2017.	Communication strategy: Targeted offer. Identification process: Each patient receiving an opioid prescription was screened for risk of opioid misuse and risk of accidental overdose based on age, concurrent medication and alcohol use, and disease states. Screening tool: Opioid Risk Tool (ORT).	Type of service/intervention: Opioid overdose education and naloxone distribution (OEND) + drug take-back + referral to community resources. Intervention details: The multicomponent statewide One Rx program included drug take-back, partial fills of opioid prescriptions, referral to community resources, naloxone education and dispensing, opioid use disorder (OUD) education, accidental overdose education, and contacting the primary healthcare provider as needed.	Time: 5 min. Setting: In-person. Materials: Welcome packet with One Rx toolkit via hardcopy binder and website; screening tool via paper, QR code, or tablet; triage tool. Model: Walk-in. Roles and processes: (Pharmacist) Patient screening and PDMP review prior to prescription pick-up, followed by education and intervention guided by a triage tool and ORT score.	Marketing: Pharmacist button advertising naloxone; window sticker; emails from Board of Pharmacy; television and newspaper interviews. Formalized policies and procedures: One Rx binder and website detailing screening, interventions, and workflow. External collaborators: North Dakota Board of Pharmacy. Pricing/reimbursement model: Funded by North Dakota Department of Human Services; USD 20 provided to pharmacies for each screening. Pharmacy awards for meeting screening benchmarks. Patient pricing model not reported.
Skoy et al., 2020b ^a^ [30]	Pharmacist authority to prescribe naloxone in North Dakota since 1 April 2017.	Communication strategy: Targeted offer. Identification process: Each patient receiving an opioid prescription was screened for the risk of opioid misuse and the risk of an accidental overdose based on age, concurrent medication and alcohol use, and disease states. Screening tool: Opioid Risk Tool (ORT).	Type of service/intervention: Opioid overdose education and naloxone distribution (OEND) + drug take-back + referral to community resources. Intervention details: The multicomponent statewide One Rx program included drug take-back, partial fills of opioid prescriptions, referral to community resources, naloxone education and dispensing, OUD education, accidental overdose education, and contacting the primary healthcare provider as needed.	Not reported in this article; see Skoy et al. 2020a.	Marketing: Not reported. Formalized policies and procedures: Not reported. External collaborators: Not reported. Pricing/reimbursement model: Naloxone product reimbursed via patient insurance or grant funds; 72% of patients received naloxone at no cost. Further details not reported.
Strand et al., 2020 ^a^ [31]	Pharmacist authority to prescribe naloxone in North Dakota since 1 April 2017.	Communication strategy: Targeted offer. Identification process: Each patient receiving an opioid prescription was screened for risk of opioid misuse and risk of accidental overdose based on age, concurrent medication and alcohol use, and disease states. Screening tool: Opioid Risk Tool (ORT).	Type of service/intervention: Opioid overdose education and naloxone distribution (OEND) + drug take-back + referral to community resources. Intervention details: The multicomponent statewide One Rx program included drug take-back, partial fills of opioid prescriptions, referral to community resources, naloxone education and dispensing, OUD education, accidental overdose education, and contacting the primary healthcare provider, as needed.	Time: Not reported in this article; see Skoy et al. 2020a. Setting: Not reported. Materials: Welcome packet with One Rx toolkit via hardcopy binder and website; screening tool via paper, QR code, or tablet; triage tool. Model: Not reported. Roles and processes: Not reported.	Marketing: Emails from Board of Pharmacy; press conference. Formalized policies and procedures: One Rx binder and website detailing screening, interventions, and workflow. External collaborators: North Dakota Board of Pharmacy. Pricing/reimbursement model: Funded by North Dakota Department of Human Services; USD 20 provided to pharmacies for each screening. Pharmacy awards for meeting screening benchmarks. Patient pricing model not reported.
Strand et al., 2019 ^a^ [34]	Pharmacist authority to prescribe naloxone in North Dakota since 1 April 2017.	Communication strategy: Targeted offer. Identification process: Each patient receiving an opioid prescription was screened for risk of opioid misuse and risk of accidental overdose based on age, concurrent medication and alcohol use, and disease states. Screening tool: Opioid Risk Tool (ORT).	Type of service/intervention: Opioid overdose education and naloxone distribution (OEND) + drug take-back + referral to community resources. Intervention details: One Rx interventions were piloted prior to scaling up statewide, including drug take-back, partial fills of opioid prescriptions, referral to community resources, naloxone education and dispensing, OUD education, accidental overdose education, and contacting the primary healthcare provider, as needed.	Time: Not reported. Setting: In-person. Materials: Opioid Misuse Risk Prevention Toolkit, including paper patient intake form, ORT, and triage tool, list of community resources, and naloxone and opioid misuse educational brochures. Model: Walk-in. Roles and processes: (Pharmacist) Patient screening, PDMP review, and “red flag” review prior to prescription pick-up, followed by education and intervention guided by a triage tool and ORT score.	Marketing: Naloxone sign and brochures. Formalized policies and procedures: Triage tool for guiding decision making. External collaborators: North Dakota Board of Pharmacy. Pricing/reimbursement model: Not reported.
Wilkerson et al., 2020 [19]	Protocol approved by local physician.	Communication strategy: Targeted off and general advertisement. Identification process: Patients at an increased risk of overdose identified by a pharmacist or intern at prescription drop-off based on criteria: high-dose opioids (> 80 MME) for chronic pain, history of OUD, and concomitant conditions (respiratory, renal, hepatic). Additionally, patient request. Screening tool: not reported.	Type of service/intervention: OEND. Intervention details: Naloxone dispensing; opioid overdose counseling.	Time: Not reported. Setting: In-person. Materials: Patient naloxone consent form (checklist); patient naloxone education brochure created by the state pharmacy board. Model: Walk-in. Roles and processes: (Pharmacist or intern) Identify at-risk patients at drop-off. (Pharmacist or intern) Patient fills out naloxone consent form. (Pharmacist or intern) Review consent form and naloxone dosage forms. (Technician) Naloxone prescription processed using a consent form as a prescription, prescriber on protocol, and name of individual requesting naloxone. (Technician) Naloxone prescription filled. (Pharmacist) Perform final check of naloxone prescription and add counseling note. (Pharmacist or intern) Review consent form and counsel on naloxone and opioid overdose at pick-up. Initial consent form, scan into dispensing system, and file with prescriptions.	Marketing: Naloxone sign outside pharmacy. Formalized policies and procedures: Program designed at corporate/clinical team level and rolled out to pharmacies with a “best practices” document. External collaborators: Not reported. Pricing/reimbursement model: Not reported.
Hines et al., 2020 [9]	West Virginia standing order.	Communication strategy: Universal offer. Identification process: All patients receiving a buprenorphine prescription. Screening tool: not reported.	Type of service/intervention: OEND. Intervention details: Patients receiving buprenorphine recruited for OEND. OEND included naloxone dispensing, education on recognizing opioid overdose, identifying opioid medications, and where to obtain naloxone.	Time: 5–20 min (10 min on average). Setting: In-person. Materials: Educational brochure from West Virginia naloxone protocol. Model: Walk-in. Roles and processes: (Pharmacy staff) Patients identified at prescription drop-off or pick-up and asked about interest in OEND. Interested patients moved to private counseling area. (Pharmacy resident) Opioid overdose education provided while waiting for buprenorphine prescription. Naloxone dispensed subsequent to obtaining a prescription from the patient’s provider; standing order not available for use during study period.	Marketing: Not reported. Formalized policies and procedures: None during the study period; resident developed business plan post-program (details not reported). External collaborators: Not reported. Pricing/reimbursement model: Naloxone product reimbursement via patient insurance.
Sexton et al., 2019 [32]	Statewide standing order.	Communication strategy: Targeted offer. Identification process: Clinical flag placed in the dispensing system to alert the pharmacist to speak with the patient at pick-up if patients had (based on CDC guidelines): an opioid prescription in the past 30 days; opioid prescription lasting ≥ 5 days; greater than or equal to 50 morphine milligram equivalents per day; concurrent benzodiazepine and opioid use; fentanyl patch greater than or equal to 25 mg/hour; and documented or verbal history of overdose or substance use disorder. Screening tool: not reported.	Type of service/intervention: OEND. Intervention details: One pharmacy implemented a standardized team-based approach (intervention); one pharmacy used the standard of practice (control). OEND included naloxone dispensing and education on opioid risks and naloxone benefits.	Time: Not reported. Setting: In-person. Materials: List of opioids at drop-off station; one-page instruction sheet at each workstation using colored paper; MME conversion chart; naloxone eligibility checklist; educational handout for patients. Model: Walk-in. Roles and processes: (Pharmacist, student pharmacist, technician) Identify naloxone-eligible patients at drop-off. Perform profile search and MME calculations. (Pharmacist) Verify that the patient meets naloxone eligibility. (Pharmacist) Place “hard stop” in dispensing software to alert the pharmacist to recommend naloxone. (Pharmacist) Recommend naloxone and provide education on opioid risks and naloxone benefits. Provide educational handout.	Marketing: Not reported. Formalized policies and procedures: Standardized one-page instruction sheet describing workflow steps posted at each station. External collaborators: Not reported. Pricing/reimbursement model: Not reported.
Teeter et al., 2021 [14]	Statewide standing order.	Communication strategy: Targeted offer (proactive) and general advertisement (passive). Identification process: (Proactive) Patients flagged in the pharmacy dispensing software if at a high risk of an overdose based on CDC guidelines (≥50 morphine milligram equivalents (MME) per day) or concurrent benzodiazepines, muscle relaxers, or sedative hypnotics. (Passive) Warning sticker on all opioid prescription vial caps dispensed; posters in the pharmacy rotated weekly. Screening tool: not reported.	Type of service/intervention: OEND. Intervention details: Naloxone dispensing and education on opioid overdose risk and naloxone using a conversation guide.	Time: Not reported. Setting: In-person. Materials: Posters derived from the MOON study [38]; counseling guide with “conversation starters;” opioid overdose education pamphlet; naloxone education pamphlet with video training links; generic naloxone demonstration kit. Model: Walk-in. Roles and processes: (Pharmacist) Provide opioid overdose education using a pamphlet and conversation guide. (Pharmacist) Recommend naloxone. (Pharmacist) Process naloxone prescription using the patient’s insurance to determine the cost. (Pharmacist) Dispense naloxone and provide education using a naloxone pamphlet or demonstration kit.	Marketing: Posters in the pharmacy rotated weekly. Formalized policies and procedures: Not reported. External collaborators: Not reported. Pricing/reimbursement model: Study funded by the UAMS Translational Research Institute and National Center of Advancing Translational Sciences. Naloxone product reimbursement via patient insurance.
Santa et al., 2021 [33]	Statewide standing order.	Communication strategy: Universal offer. Identification process: Pharmacy-specific protocols used for recommending naloxone to all patients prescribed opioids (details not reported). Screening tool: formal screening tool mentioned, but details not reported.	Type of service/intervention: OEND. Intervention details: Screening, brief intervention, and referral to treatment (SBIRT). Brief intervention included counseling regarding opioid overdose and naloxone using motivational interviewing (MI) and naloxone dispensing.	Time: Not reported. Setting: In-person. Materials: SBIRT proficiency checklist; workflow outline using the “A3” format. Model: Not reported. Roles and processes: (Pharmacist) At least one pharmacist per site served as a program champion. (Champion) Check other pharmacists’ SBIRT abilities using a checklist. (Champion) Monitor naloxone dispensing using a workflow outline each week.	Marketing: Not reported. Formalized policies and procedures: Formal naloxone dispensing policies and procedures developed by a pharmacist site champion, specific to each pharmacy (details not reported). Workflow outline for ensuring protocol fidelity. External collaborators: Not reported. Pricing/reimbursement model: Study funded by the Pennsylvania Commission on Crime and Delinquency. Patient pricing model not reported.

^a^ Multiple articles describing implementation processes from the same study or a related pilot study (One Rx program).

**Table 6:** Programmatic outcomes.

Study	Uptake and Delivery	Intervention Outcomes	Satisfaction
Akers et al., 2017 [10]	Number of participants reached: Held 27 group events with 5–350 participants each. Trained 1400 people about naloxone overall.	Amount of naloxone recommended/dispensed: Dispensed 234 naloxone kits from the pharmacy. Dispensed 505 naloxone kits via partnership with local county psychologist. Other OCN program-specific outcomes: 20.2% rescue rate reported from naloxone kits dispensed by the pharmacy.	Patient satisfaction: Anecdotally reported that the program was viewed favorably. No further details reported. Provider satisfaction: Anecdotally reported that the program was viewed favorably. No further details reported.
Cochran et al., 2019 [27]	Number of participants reached: 387 patients identified, 314 screened, 32 consented to participate (BMI-MTM *n* = 15, SMC *n* = 17). A total of 93% retained at 3 months.	Amount of naloxone recommended/dispensed: Not reported. Other OCN program-specific outcomes: After 3 months, the odds of opioid misuse were lower in the BMI-MTM group compared to those in the SMC group (AOR = 0.13; 95% CI = 0.05–0.35, *p* < 0.001).	Patient satisfaction: 92.4% of BMI-MTM participants satisfied with the program, with a mean rating of 4.2 out of 5. Provider satisfaction: Not reported.
Manzur et al., 2020 [28]	Number of participants reached: 19 sessions delivered, 11 patients assessed (1–2 sessions per patient).	Amount of naloxone recommended/dispensed: Naloxone prescribed to 58% of patients. Other OCN program-specific outcomes: Pharmacists identified opioid/pain medication-related problems most frequently surrounding the need for additional treatment (84%). A total of 74% of recommendations were for additional lab tests, and 58% were related to adjusting the dose of opioid/pain medication.	Patient satisfaction: Not reported. Provider satisfaction: Not reported.
Skoy et al., 2020a [29]	Number of participants reached: 1700 patients screened, and 240 pharmacists and 41 technicians trained across 30 pharmacies.	Not reported.	Not reported.
Skoy et al., 2020b [30]	Number of participants reached: 2716 patients screened; 1371 potentially at risk for opioid overdose or misuse.	Amount of naloxone recommended/dispensed: Naloxone recommendations accepted by 5.81% of patients. Other OCN program-specific outcomes: The naloxone acceptance rate was higher in those with more overdose risk factors (e.g., zero risk factors = 1.18%, five risk factors = 17.09%; *p* < 0.05).	Not reported.
Strand et al., 2020 [31]	Number of participants reached: Sixty-three pharmacies enrolled in the program; thirty actively participated. A total of 1685 patients screened.	Amount of naloxone recommended/dispensed: Not reported. Other OCN program-specific outcomes: Reach: patients taking opioid who were screened (16.9%). Efficacy: patients at a high risk of overdose/misuse given intervention (97.1%). Adoption: Pharmacies in North Dakota enrolled in the program (45%). Implementation: Pharmacies that enrolled and participated by conducting five or more screenings (44.8%). Maintenance: Pharmacies that continued participating in the program at 3 months (80%).	Not reported.
Strand et al., 2019 [34]	Number of participants reached: 107 patients screened.	Amount of naloxone recommended/dispensed: 43 recommended, 5 prescribed, 3 dispensed. Other OCN program-specific outcomes: 30% and 25% of patients screened were at risk of opioid overdose and misuse, respectively. “Red flags” detected in 23% of screenings.	Not reported.
Wilkerson et al., 2020 [19]	Number of participants reached: 1000 pharmacists and interns across 102 pharmacies.	Not reported.	Not reported.
Hines et al., 2020 [9]	Number of participants reached: 52 patients enrolled.	Amount of naloxone recommended/dispensed: 15 naloxone kits dispensed after intervention; 2 dispensed before intervention. A 400% increase in dispensing. Other OCN program-specific outcomes: After counseling, more patients believed that they should have naloxone on hand compared to before receiving counseling (40.4% compared to 15.4%, *p* < 0.001).	Not reported.
Sexton et al., 2019 [32]	Number of participants reached: 39 patients.	Amount of naloxone recommended/dispensed: 11 naloxone kits dispensed by the intervention group during the study period compared to 3 before the study period. A 367% increase in dispensing. Other OCN program-specific outcomes: Not reported.	Patient satisfaction: Anecdotally reported that two patients used the naloxone dispensed to them and told the pharmacist they were happy they received it. Provider satisfaction: Not reported.
Teeter et al., 2021 [14]	Number of participants reached: 148 patients approached; 130 consented to participate.	Amount of naloxone recommended/dispensed: 44 naloxone kits dispensed by the intervention pharmacy; 0 dispensed by control pharmacies. Other OCN program-specific outcomes: Naloxone dispensed to 15.8% of patients receiving ≥50 MME and 36.7% of patients with concomitant medications that increased the risk of opioid overdose.	Patient satisfaction: Not reported. Provider satisfaction: Upon post-program interviews, pharmacy personnel indicated that the service was feasible, acceptable, and appropriate.
Santa et al., 2021 [33]	Number of participants reached: 2082 patients. A total of 24 pharmacists recruited; 22 pharmacists participated.	Amount of naloxone recommended/dispensed: 1160 naloxone kits recommended; 430 naloxone kits dispensed after the intervention (compared to 180 before the intervention). Other OCN program-specific outcomes: The amount of naloxone dispensed varied by the pharmacy type (independently owned *n* = 245, corporately owned *n* = 185).	Not reported.

## Data Availability

Not applicable.

## Supplementary Materials

The following supporting information can be downloaded at: https://www.mdpi.com/article/10.3390/pharmacy11030099/s1, Table S1: Opioid Counseling and Naloxone Service Implementation Resources and Tools.
