# Supplementary material for: Organizational Readiness to Implement Community Pharmacy-Based Opioid Counseling and Naloxone Services: A Scoping Review of Current Practice Models and Opportunities
Source: Pharmacy (Basel). 2023 Jun 11;11(3):99. doi: 10.3390/pharmacy11030099 (PMC10302283; doi:10.3390/pharmacy11030099)
Supplement: Supplementary file 1 [file pharmacy-11-00099-s001.zip › pharmacy-2410434-supplementary.pdf]

Supplementary Material

Table S1. Opioid counseling and naloxone service implementation resources and tools.

**Resource / tool name**	**Resource / tool content and purpose**				
	**Opioid misuse screening**	**Patient education materials**	**Pharmacist and provider education**	**Opioid prescribing guidelines**	**Pain management assessment**
Substance Abuse and Mental Health Services Administration (SAMHSA) toolkit [36]		X	X		
Prescribe to Prevent [37]		X	X		
Maximizing OpiOid Safety with Naloxone (MOON) study [38]		X			
One Rx Program [42]		X	X		
Interagency Guideline of Prescribing Opioids for pain from the Washington State Agency Medical Directors Group [39]			X	X	
Centers for Disease Control and Prevention (CDC) Clinical Practice Guidelines for Prescribing Opioids for Pain [35]			X	X	
Prescription Opioid Misuse Index (POMI) [40]	X				
Opioid Risk Tool [ORT] [41]	X				
SBIRT framework (screening, brief intervention, and referral to treatment) [43]	X				
Pain, Enjoyment, General Activity (PEG) tool [44]					X
